# Aging induced decline in T-lymphopoiesis is primarily dependent on status of progenitor niches in the bone marrow and thymus

**DOI:** 10.18632/aging.100487

**Published:** 2012-10-08

**Authors:** Liguang Sun, Robert Brown, Shande Chen, Qichuan Zhuge, Dong-Ming Su

**Affiliations:** ^1^ Department of Molecular Biology and Immunology, University of North Texas Health Science Center at Fort Worth, Fort Worth, TX 76107, USA; ^2^ Department of Biostatics, University of North Texas Health Science Center at Fort Worth, Fort Worth, TX 76107, USA; ^3^ Institute of Translational Medicine, Norman Bethune First Hospital, Jilin University, Changchun City, 135505, China; ^4^ University of Texas Health Science Center at Tyler, Tyler, TX 75708, USA; ^5^ The First Affiliated Hospital, Wenzhou Medical College, Wenzhou city, Zhejiang Province, 325000, China

**Keywords:** Hematopoiesis, bone marrow, T-lineage cells, niche cells, microenvironment, thymic stromal cells, aging, competitive repopulation

## Abstract

Age-related decline in the generation of T cells is associated with two primary lymphoid organs, the bone marrow (BM) and thymus. Both organs contain lympho-hematopoietic progenitor/stem cells (LPCs) and non-hematopoietic stromal/niche cells. Murine model showed this decline is not due to reduced quantities of LPCs, nor autonomous defects in LPCs, but rather defects in their niche cells. However, this viewpoint is challenged by the fact that aged BM progenitors have a myeloid skew. By grafting young wild-type (WT) BM progenitors into aged IL-7R^−/−^ hosts, which possess WT-equivalent niches although LPCs are defect, we demonstrated that these young BM progenitors also exhibited a myeloid skew. We, further, demonstrated that aged BM progenitors, recruited by a grafted fetal thymus in the *in vivo* microenvironment, were able to compete with their young counterparts, although the *in vitro* manipulated old BM cells were not able to do so in conventional BM transplantation. Both LPCs and their niche cells inevitably get old with increasing organismal age, but aging in niche cells occurred much earlier than in LPCs by an observation in thymic T-lymphopoiesis. Therefore, the aging induced decline in competence to generate T cells is primarily dependent on status of the progenitor niche cells in the BM and thymus.

## INTRODUCTION

The progressive degeneration and functional decline of cells, tissues, and organs that accompany aging are unavoidable. To date, the most rigorous studies on aging have been conducted on the T-lymphoid immune organs, primarily comprising the reservoir for hematopoietic stem cells (HSCs) – the bone marrow (BM), and the immature T-cell cradle and educators – the thymus gland. These organs are all comprised of two main cellular components of different origins: lympho-hematopoietic — immature and mature T cells and non-lympho-hematopoietic — stromal or niche cells. These two cellular components interact with each other (lympho-stromal interaction) via soluble factors and cell-to-cell contacts that initiate gene expression to promote development of T-cell immune system and control the process of T-cell immune system aging. Although both lympho-hematopoietic and non-lymphohematopoietic components have been found not to be truly immortal, they are unlikely to develop age-related intrinsic defects at the same rate or show simultaneous mortality [1]. It is debatable if aging-induced failure to generate T cells, characterized by age-related thymic involution that reduces the naïve T-cell pool, is due to intrinsic defects in the lympho-hematopoietic progenitor/stem cells (LPCs or HSCs) themselves, or is adversely regulated by intrinsic defects of their niche cells, including mesenchymal stem cell-derived osteoblasts in the BM [2-4] and thymic epithelial cells (TECs) in the thymus [1, 5, 6]. It is important to clarify this clinically relevant issue so that selection of the right target(s) and development of proper strategies to rejuvenate the aged immune system can be implemented. Such an approach will hopefully delay and/or treat the onset of age-related immune deficient diseases.

It has been well documented that aging-induced decline of lymphocytes is not due to reduction of quantities in BM progenitors, because in mice, quantities of HSCs within BM are generally increased, instead of decreased (Six reports summarized in a recent review [7] and our own observation [1]), while in human, aged BM progenitor numbers are either decreased [8] or increased [9]. At least, in mice, quality, rather than quantity, of aged BM progenitors, is considered to contribute to decreased lymphopoiesis with aging. One of main changes of aged BM progenitor characteristics is a myeloid-skew (alteration in HSC differentiation), i.e. aged BM progenitors produce greater numbers of myeloid than lymphoid lineage cells compared to young BM progenitors [3, 10-12]. This seems that aging of the T-lymphoid system occurs due to primary and intrinsic changes in LPCs/HSCs. However, recently a few but intriguing studies have reported that the primary and dominant cause of lymphoid system aging may be attributed to their endogenous microenvironment, constituted by stem-cell niche cells (i.e. stromal cells), in the BM [2, 3] and thymus [1, 6]. Deterioration of these microenvironments induces LPC/HSC defects, and ultimately leads to a failure of the entire T-cell generation system.

The notion of aging-induced defects in the stem-cell niche rather than in the stem cells themselves, leading to system wide failure has been recently recognized in many systems, such as aging in oocytes/ovary [13-15], sperm/testis [16, 17], and muscles [18, 19]. However, this scenario has not been widely accepted in T-lymphoid system aging. This is in part due to the inadequate understanding of the divergent down-regulated lymphoid and up-regulated myeloid gene expression profile in aged BM progenitors [20], and the myeloid-biased cellular differentiation of aged BM progenitors [10, 12], despite the argument that these may arise from the loss or expansion potential from any particular niche [21, 22]. Therefore, it is of utmost importance to determine whether old niches in the BM and/or thymus can directly affect competence of young LPCs to generate T cells. Furthermore, it is important to delineate the interactions between LPCs and their niche via integrative analysis of both LPCs and their niche cells in the same developmental microenvironments.

A syngeneic bone marrow transplantation (BMT), where old BM progenitors are introduced into a young mouse environment and vice versa, is a common *in vivo* developmental setting to address this issue [23]. In this respect, BMT into irradiated host mice [10] was found to produce inaccurate results due to radiation-induced damage to stromal cells [12]. Likewise the use of non-irradiated wild-type (WT) host mice presented a different set of problems as host niches are predominantly occupied leaving only 0.1-1.0% of the HSC niches available for engraftment [24]. However, hosts with lympho-hematopoietic genetic mutation but possessing normal genetic (WT-equivalent) niche cells have been demonstrated to have an improved durability in stem cell engraftment for studying stem cell function in non-irradiation hosts [24, 25]. Although non-hematopoietc stromal cells in these lympho-hematopoietic deficient hosts are at pre-developmental state, due to lack of lympho-stromal crosstalk, these pre-developmental stromal cells will be soon re-programmed to provide normal environment for supporting hematopoietic cell development, as soon as normal HSCs are introduced in the system and participate in the crosstalk. The IL7R gene knockout mouse is one of these kind hosts. Therefore, conducting a syngeneic BMT in IL7R^−/−^ host mice [26] instead of WT host mice, circumvents the problem of predominantly occupied host niches [27].

Purified subpopulations of T-cell precursors used in *in vitro* culture settings, such as fetal thymic organ culture (FTOC) [28] and OP9-DL1 monolayer culture [12, 29] is also a developmental setting to address the effects of aging on lympho-stromal interactions. However, *in vitro* manipulation of cells, such as flow cytometric sorting, may cause a defect in old LPCs, such as transplantability [30] and/or decreased adhesion to stroma [31]. In addition, since the potential thymus-seeding T-lymphocyte progenitors are complex and undefined [32], any single subpopulation isolated by cell sorting may not reflect the full differentiation potential of natural thymus-seeding LPCs. Therefore, recruitment of natural thymus-seeding LPCs *in vivo* using kidney capsule transplantation (KCT) of fetal thymic lobes was designed to overcome the above pitfalls [1].

In this study, we have circumvented the issues described above by developing several novel and comprehensive *in vivo* and *in vitro* models, such as using IL7R^−/−^ [26] host mice for BMT, designing a cross-KCT (cKCT) model to provide the same microenvironment for competition, and using KCT-recruited young and old LPCs for a competitive co-culture on an OP9-DL1 stromal monolayer [29], to delineate the interactions between hematopoietic stem and their niche cells.

## RESULTS

### Age-related alterations in the myeloid vs. lymphoid differentiation of young BM progenitors may be due to the age of the host BM niches

It is well known that aged BM progenitors follow myeloid-biased differentiation [10, 12]. But it is largely unknown whether this is due to primary alteration in BM progenitors themselves or their niches (endogenous microenvironment). We asked what differentiation profile would be if young BM progenitors replace aged BM progenitors and stay in aged niches. This stimulated our efforts to obtain direct evidence whether the aged niches are responsible for the differentiation.

The OP9-DL1 stromal cell monolayer has been previously used for successful development of a singlesource of BM progenitors [33]. In this culture setting, LPCs from old BM differentiated fewer T-lineage cells than myeloid lineage cells compared to LPCs from young BM [12]. Based on this finding, we developed a BMT combining OP9-DL1 system.

**Figure 1:**
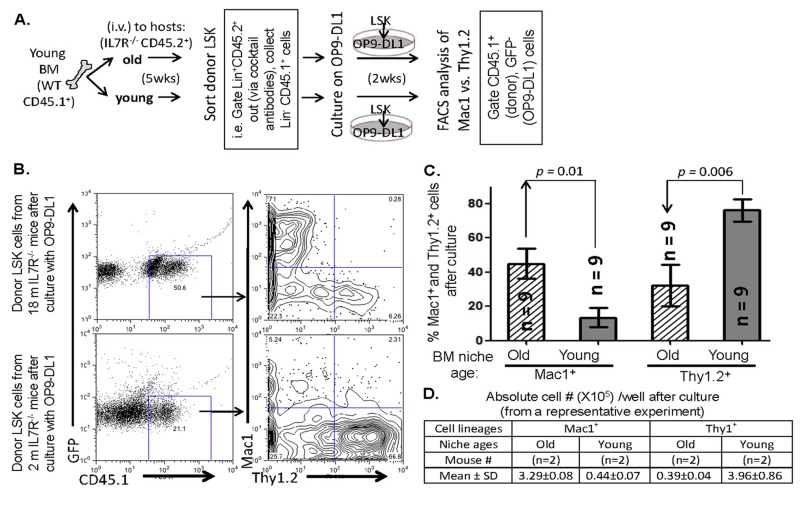
Comparison of the influence of BM niche age on differentiation profile of T-lineage vs. myeloid lineage choice **(A)** Schematic workflow of the BM-niche age influence assay. **(B)** A representative flow cytometry analysis shows donor congenic marker gates (left panels), and profiles of T-lineage (Thy1.2^+^) vs. myeloid-lineage (Mac1^+^) cells derived from old (18 months old) and young (2 months old) IL7R^−/−^ host BM niches after culture on OP9-DL1 stromal cell monolayer (right panels). These cells were originally from the same pool of young WT BM progenitors. **(C)** A summary of panel B in % myeloid-lineage cells (left) and % T-lineage cells (right) derived from old (striped bar) and young (grey bar) IL7R^−/−^ host BM niches. Data show Mean ± SEM in all bar graphs, n = IL7R^−/−^ host mouse number. **(D)** A representative culture result of Panel A shows absolute cell numbers (Mean ± SD) of myeloid cells vs. T-lineage cells derived from old or young IL7R^−/−^ host BM niche-modulated young WT donor BM progenitors (~2000 sorted LSK cells loaded per well) after 14 days in culture on OP9-DL1 stromal cell monolayer (n = IL7R^−/−^ host animal #).

By taking advantage of IL7R^−/−^ mouse WT-equivalent stromal cells and vacant niches [24, 25, 27], we first transplanted the CD45.1^+^ young BM cells from the same pool into either young (~2-month-old) or old (22-month-old) non-irradiated CD45.2^+^ IL7R^−/−^ recipient host mice. Five weeks after the BMT, we checked donor (CD45.1^+^) BM progenitor constituted BM cell numbers in the hosts (CD45.2^+^), and found that the reconstituted donor BM cells did not have significant quantitative difference in the grafted young and aged hosts (Supplemental Figs. S3A and C left panel, and data not shown). And then, we sorted donor (CD45.1^+^) LSK (lineage- and host-CD45.2 negative, Sca-1^+^, and c-Kit^+^) cells (Supplemental Fig. S1) from the grafted host BM, plated the same number of sorted LSK cells from the grafted young and old hosts on the OP9-DL1 stromal cell monolayers and co-culture them as previously described [12] and as shown in workflow diagram (Fig. 1A). After two weeks in culture, the young LPCs modulated by an old BM microenvironment differentiated fewer T-lineage cell than myeloid lineage cells, similar to old LPCs [12], when they were compared to young LPCs from a young microenvironment (Figs. 1B-D). Our results indicate that differentiation of the skew of T-lymphoid to myeloid lineage is associated with the stem cells modulated (maybe receiving different signals) in BM niches of different ages.

### Aged LPCs can compete with their young counterparts in a cKCT setting, although they cannot do so in a conventional BMT setting

**Figure 2:**
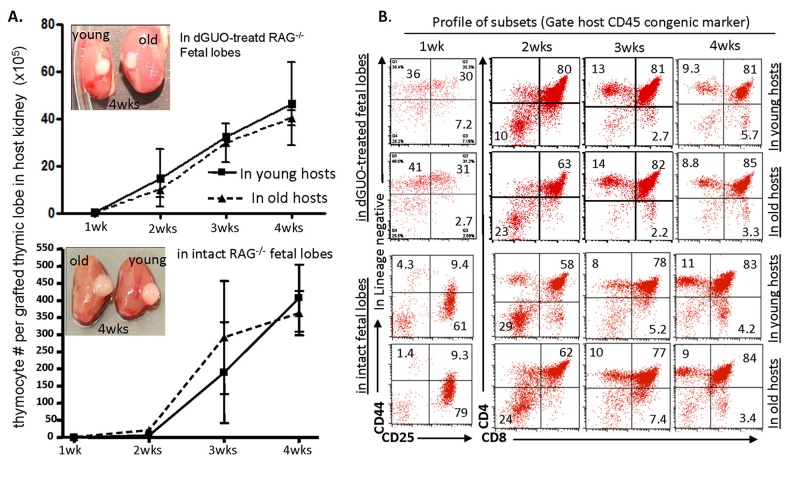
Competence of thymic T-lymphopoiesis from aged- and young-LPCs in repopulation of grafted fetal thymic lobes *in vivo* in a time course manner (**A**) Thymocyte number in dGUO-treated (top panel) or intact (bottom panel) grafted fetal thymic lobes, at various weeks after transplanted under the kidney capsules of young (~2 months) and old (20 - 22 months) WT mice. The images shown are representative results of grafted thymic lobe size in the hosts? kidney capsules. Each data point (triangle or square) represents 2-3 host mice. (**B**) A representative result shows differentiation profiles (CD44 vs. CD25 at one-week time point, and CD4 vs. CD8 at all other longer time points) of thymocytes from grafted fetal thymic lobes under the young and old kidney capsules.

It is well known that co-transplantation of young and old BM progenitor cells into a young WT host in conventional BMT, old BM progenitor cells could not compete with their young counterparts in generating T-lineage cells, no matter whether the hosts are irradiated [10] or non-irradiated [12]. The conventional BMT has an *in vitro* manipulation process of BM progenitors. Evidence showed that when aged BM progenitors stay *in vitro*, they display decreased adhesion to stroma [31]. We asked whether aged BM progenitors recruited *in vivo* by a grafted fetal thymus are able to compete with their young counterparts, because when we examined *in vivo* repopulation in the fetal thymus with aged LPCs over 1 to 4 weeks in a KCT model and in a time-course manner [1, 34], we found that LPCs from old BM were able to generate the same number of thymocytes (Fig. 2A) with similar differentiation profiles as young BM progenitor did (Fig. 2B). The longer the progenitors remained in the fetal microenvironment, the less distinction was observed between the young and old groups as defined by CD4 vs. CD8 differentiation profiles (Fig. 2B). These results were independent of whether cells matured in dGUO-treated or intact fetal thymus at any of the sampled time points. This encourages us to develop a novel *in vivo* competitive method based on KCT.

**Figure 3:**
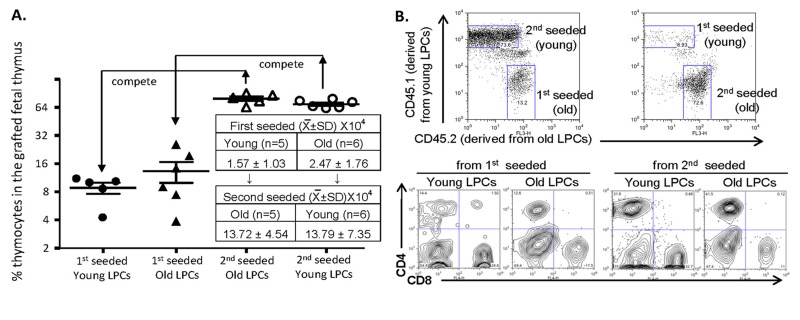
Comparison of competence for thymic T-lymphopoiesis from aged- and young-LPCs’ in competitive repopulation of grafted fetal thymus (**A**) Results of competitive repopulation of grafted fetal thymus by “young and old” or “old and young” natural thymus-seeding cells from the first and second hosts. Left panel shows % T-lineage thymocytes (gated on DP, CD4^+^ and CD8^+^ SPs) in the grafted thymus naturally seeded by young (~2 month old, circles) and old (22 months old, triangles) BM progenitors in the different seeding orders (initial seeding: filled circles or triangles; subsequent/second seeding: open circles or triangles). Each triangle or circle represents one animal. An unpaired Student's *t*-test shows *p* > 0.05 (no significant). The table in the A panel shows absolute cell numbers per grafted lobe (each host mouse was grafted with 2-3 fetal thymic lobes, n = animal number). (**B**) A representative result of differentiated CD4^+^ and CD8^+^ T cells from the grafted fetal thymic lobes (bottom panels). The thymocytes are derived from first and second seeded young- and old-BM progenitors, identified by CD45.1 and CD45.2 congenic markers (top panels).

We, therefore, developed a cross-KCT (cKCT) model (Supplemental Fig. S2) by transplanting dGUO-treated CD45.1^+^CD45.2^+^ RAG^−/−^ fetal thymic lobes, which are a good *in vivo* model to accumulate WT LPCs [1, 34], into young CD45.1^+^ or old CD45.2^+^ mice (first hosts). Five days after the first host KCT, cKCT was performed by transplanting the fetal thymic lobes carrying seeded LPCs from the first hosts into age-reversed and CD45-subtype-reverseed second hosts to recruit LPCs from the second hosts for additional 5 days. The lobes containing “young and old” or “old and young” LPCs were then subjected to FTOC for one week. The later seeded LPCs from the second hosts of either young or old mice became a dominant population in the lobes after FTOC (Fig. 3A, open triangles and open circles). The KCT young LPCs were then compared with KCT old LPCs (both from the first hosts), while the cKCT young LPCs were compared with the cKCT old LPCs (both from the second hosts). In this *in vivo* competitive experiment we found no significant difference in T-lineage cell numbers generated (Fig. 3A) and in the differentiation of CD4^+^ and CD8^+^ single positive T cells (Fig. 3B) derived from young and old LPCs in the fetal thymic lobes. Taken together, our results indicate that the old LPCs did not lose their competence for T-cell generation and were equal in this respect with young LPCs when allowed to naturally seed the thymus but were not subjected to *in vitro* manipulation.

### Aged BM progenitors, recruited by grafted fetal thymic lobes *in vivo*, compete with young BM progenitors even subjected subsequently to in vitro culture

**Figure 4:**
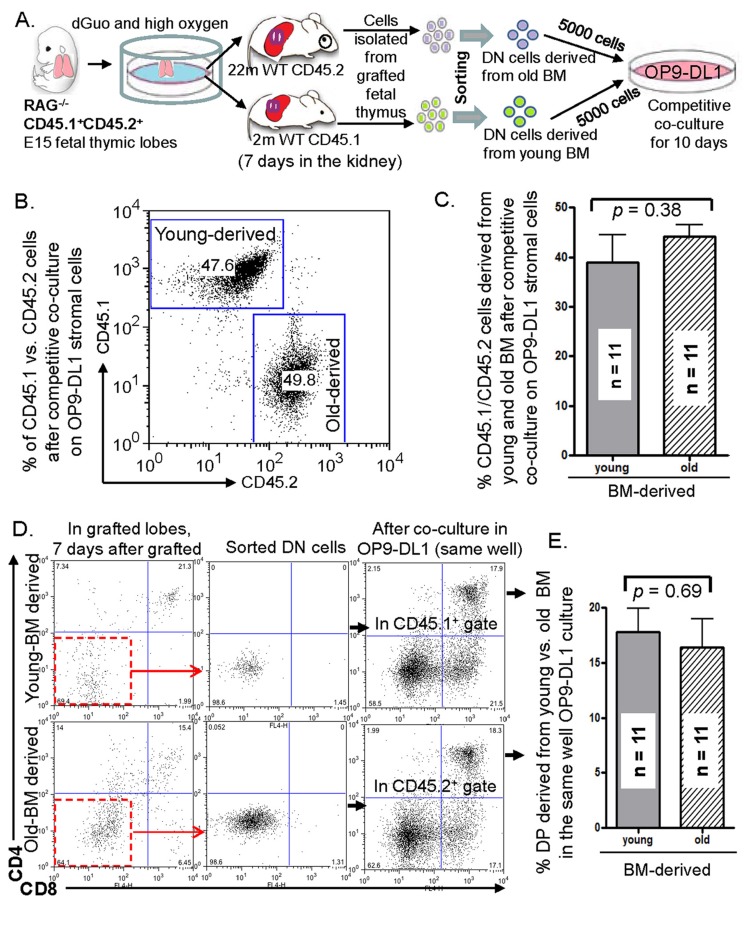
*In vitro* competition between aged and young LPCs accumulated by grafted fetal thymic lobes *in vivo* under old or young mouse kidney capsules **(A)** Schematic workflow of the comprehensive competitive culture assay showing the recruitment of old and young natural thymus-seeding cells *in vivo* to the *in vitro* competitive co-culture on OP9-DL1 monolayer stromal cells. **(B)** A representative result of % T-lineage cells derived from old (CD45.2^+^) and young (CD45.1^+^) thymus-seeding LPCs after competitive co-culture on OP9-DL1 stromal cell monolayer. **(C)** A summary of % T-lineage cells derived from old (CD45.2^+^) and young (CD45.1^+^) thymus-seeding LPCs after competitive co-culture on OP9-DL1 stromal cells. **(D)** A representative flow cytometry dot-plot shows CD4 vs. CD8 profile of T-lineage cells from the grafted fetal thymic lobes 7 days after KCT (left panels); purification of DN cells after negative-selection with beads (middle panels); and CD4 vs. CD8 profile of T-lineage cells after competitive co-culture on OP9-DL1 stromal cells (right panels). **(E)** A summary of % CD4^+^CD8^+^ (DP) cells derived from old (CD45.2^+^) and young (CD45.1^+^) thymus-seeding LPCs after competitive co-culture on OP9-DL1 stromal cells. Data in C and E panels show mean ± SEM, n = competitive co-culture wells; total host animal number is 5 young and 5 old WT mice. The experiment was conducted 5 times (i.e. 5 FTOC, 5 sorts, and 5 cultures).

We were curious if natural thymus-seeding aged BM progenitors, accumulated *in vivo* during fetal thymus KCT, can compete in T-cell generation with their young counterparts in an *in vitro* culture model. To test this, we designed and performed competitive cultures on an OP9-DL1 stromal cell monolayer (workflow in Fig. 4A). After young CD45.1^+^ and old CD45.2^+^ natural thymus-seeding cells were recruited *in vivo* from dGUO-treated CD45.1^+^CD45.2^+^ RAG^−/−^ fetal thymic lobes in KCT of young CD45.1^+^ and old CD45.2^+^ host mice for 7 days, we selected CD4^−^8^−^ DN population from the lobes, and loaded the DN cells at equal numbers on the same monolyayer co-culture for 10 days. The results showed that LPCs from young- and old-sources had the capacity to generate equal numbers and proportions of T-lineage cells (Figs. 4B and 4C) and an equal % of CD4^+^CD8^+^ double positive (DP) thymocytes (Figs. 4D and 4E). It suggests two possible scenarios: 1) Aged BM contains mixed population of defective and functional progenitors, and only the functional progenitors can naturally seed the fetal thymus. Therefore they can compete with young LPCs; 2) The competence of T-cell generation from the natural thymus-seeding old BM progenitors can be recovered to the young progenitor levels after the exposure to the fetal thymic microenvironment for a certain period of time.

### Aging of LPCs though unavoidable occurs more slowly than aging of their niche cells

Although we did not find a significant reduction in the capacity of aged LPCs to generate T-lineage cells either in the young thymic microenvironment (Figs. 2 and 3), or after “rejuvenation” by a young thymic microenvironment (Fig. 4), these aged LPCs are not immortal. Aging, after all, induces stress in BM progenitors, as is evident from conventional BMT [12]. To determine at which age and how quickly LPCs show a marked increase in transplantation stress, associated with the age-related disability of their BM niche cells (osteoblastic cells in the BM) and thymic niche cells (TECs in the thymus), we designed an experiment, in which competence of thymic T-lymphopoiesis associated with LPC age or niche-cell age was observed in the recipient thymus.

**Figure 5:**
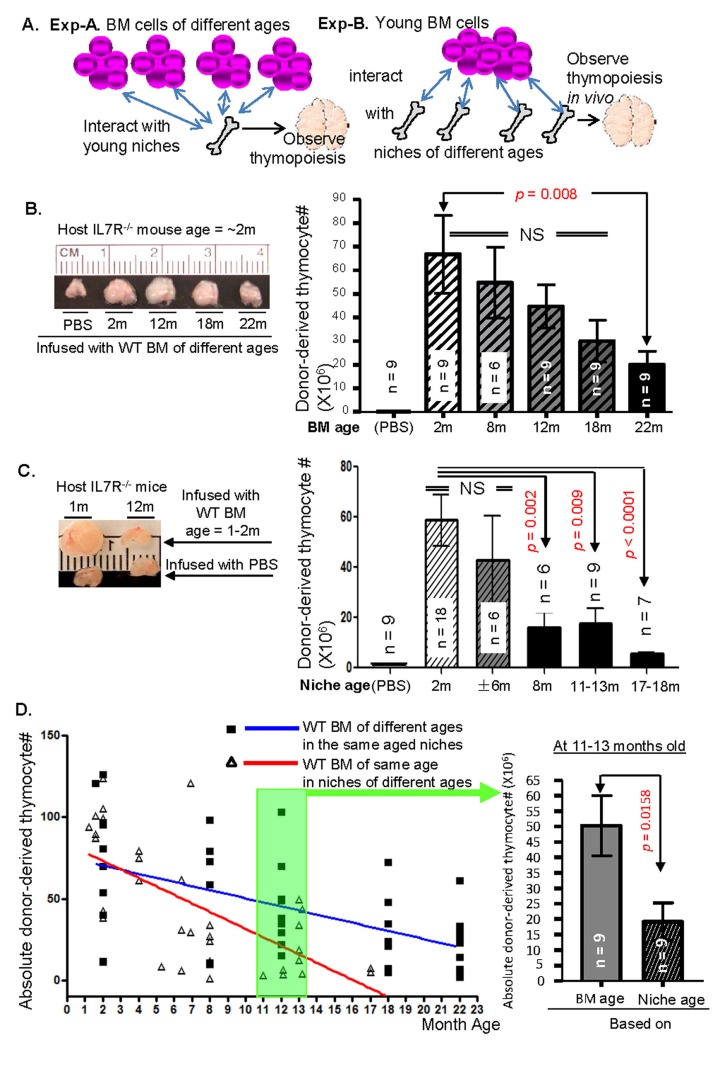
Comparing competence of thymic T-lymphopoiesis associated with BM progenitor ages or their niche ages in repopulating IL7R^−/−^ host mouse thymus in a BMT microenvironment **(A)** A schematic diagram of workflow. **(B)** Left panel shows the gross appearance of young IL7R^−/−^ mouse thymus size from a representative experiment, 5 weeks after infusion with PBS, or equal numbers of 2-, 8-, 12-, 18-, or 22-month-old WT BM cells. Right panel shows a summary of total thymocyte number in young IL7R^−/−^ host mouse thymus, derived from donor WT mouse BM cells of different ages. **(C)** Left panel shows the gross appearance of 1- (left column) and 12-month-old (right column) IL7R^−/−^ mouse thymus size from a representative experiment, 5 weeks after transplantation with equal numbers of ~2-month-old WT BM cells (top row) or PBS (bottom row). Right panel shows a summary of total thymocyte number derived from young donor WT mouse BM cells in IL7R^−/−^ host niches of different ages. **(D)** Left panel shows the linear regression of thymocyte number derived from donor WT BM cells of different ages in young IL7R^−/−^ host niches (blue line, Exp-A) and from young donor WT BM cells in IL7R^−/−^ host niches of different ages (red line, Exp-B). Test for equal slopes for the blue (slope −3.72 ~ −1.28) and red (slope −7.23 ~ −3.97) gives a (2-sided) p-value of 0.016 (significantly different). Right panel shows donor-derived thymocyte numbers from 11-to-13-month-old donor WT BM cells in young IL7R^−/−^ host niches (left bar) and young donor WT BM cells in 11-to-13-month-old IL7R^−/-^ host niches (right bar). Data show mean ± SEM in all bar graphs, n = IL7R^−/−^ host animal number, each triangle and square in C represents one animal. Experiments were repeated over 5 times.

When WT mouse BM cells are transplanted into IL7R^−/−^ host mice, they can restore the small thymus size of IL7R^−/−^ mouse to normal size and develop normal number of mature T cells [27]. This is because IL7R^−/−^ host mice have WT-equivalent niche cells in the BM and thymus, and these niches can accept and support development of WT BM progenitors. The process is dependent upon lympho-stromal interaction between donor LPCs and host niches. Based on this, we designed a set of experiments (Experiments A and B, workflow is shown in Fig. 5A). In experiment-A, WT BM cells, isolated from 2-, 8-, 12-, 18-, and 22-month-old WT mice were transplanted into age-mismatched young (~2-month-old) IL7R^−/−^ host mice. This allows determination of age at which the LPCs show significant loss of transplant efficacy and undergo a subsequent reduction in their ability of thymic T-lymphopoiesis in young BM and thymic microenvironments. In experiment-B, we then transplanted a pool of BM cells, isolated from young (~2-month-old) WT mice, into age-mismatched IL7R^−/−^ host mice at 2-, 6-, 8-, 12~13-, 18-months of age to provide niches of different ages. This allows us to determine the age at which the niche cells in the BM and thymus lose their ability to support LPCs in T-cell generation. Thymocyte numbers of each group were compared to those in 2-month-old group respectively in order to determine the age at which the T-lymphopoisis was significantly reduced.

Our results showed that although the competence of LPCs to generate thymic T-lymphopoiesis dynamically declined with increasing age, only when the LPCs reached 22 months of age, was the thymic T-lymphopoisis significantly reduced when compared to that of young LPCs (Fig. 5B). However, the capacity of the niche cells to support young LPC's thymic T-lymphopoiesis was significantly reduced from 8-12 months of age, much earlier than in the case of old LPCs (Fig. 5C). Comparing thymocyte numbers from Exp-A and Exp-B in the same linear regression plot, a slope of decline based on niche cell age (Exp-B) showed more steeper than the one based on BM cell age (Exp-A), and the test for equal slopes for these two groups gave a (2-sided) p-value of 0.016 (significantly different) (Fig. 5D, left panel). The thymocyte numbers generated from young LPCs supported by 11~13-month-old niche cells were significantly lower than those generated from the same aged LPCs in young niches (Fig. 5D, right panel), clearly supporting the conclusion that LPCs have longer lifespan than their niche cells on the thymic T-lymphopoisis.

Unlike peripheral lymphoid organs, such as the spleen, the thymus is not the site of executing the immunological rejection. The decrease in CD45.1^+^ WT donor-derived thymocytes with increased recipient ages in the CD45.2^+^ IL7R^−/−^ thymus is unlikely due to immunorejection, although IL7R^−/−^ recipient mice are considered incompletely immunodeficient. In order to determine whether there is an age-related immunological difference in IL7R^−/−^hosts, we checked the chimerism of the CD45.1-subtype progenitors in the CD45.2^+^ BM of different ages (Supplemental Figs. S3A and C left panel). No differences of CD45.1^+^ donor-derived BM cells were found among 2- and 8-month-old CD45.2^+^ IL7R^−/−^ hosts, whereas, 8-month-old CD45.2^+^ IL7R^−/−^ thymic niches have already exhibited a significantly reduced competence to support CD45.1^+^ donor thymocyte development (Fig. 5C, and Supplemental Figs. S3B and C right panel). By observing peripheral lymphocytes, while donor-derived numbers of splenic B cells were slightly increased (Supplemental Figs. S3B and D), donor-derived numbers (Supplemental Fig. S3D) and % (Supplemental Fig. S4) of splenic CD4^+^T cells were significantly decreased at the age of 8-12 months old, which is probably due to decreased thymic output, and it is one of reported immune system aging phenotypes [35].

## DISCUSSION

In the BM and thymus, lympho-hematopoietic and non-hematopoietic (stem-cell niche or stromal) cellular compartments tightly regulate competence for T-cell generation, while aging clearly induces defects in this competence [36]. It is still controversial whether the defect originates in the LPC/HSC itself or in the supportive microenvironment. To understand the cellular mechanism of aging in the T-cell generation it is necessary to determine which cellular compartment develops the primary/intrinsic defect. We demonstrate here that this defect originates primarily from the endogenous microenvironment, as evidenced by the fact that the aged BM niches could induce the same defects in BM progenitors from young mice (Fig. 1). Our findings also show that in mice, LPCs seem to lack an intrinsic defect prior to 20-22 months of age, (comparable to ~80-year-old humans), since they were able to repopulate the dGUO-treated and intact fetal thymic lobes *in vivo* over either short (1 week) or long (4 weeks) time period, comparable to LPCs from young mice (Fig. 2). Natural thymus-seeding LPCs from aged and young mouse BM, acquired *in vivo* by a grafted fetal thymic lobe, compete well each other to generate T-lineage cells in a common young microenvironment (Fig. 3). These natural thymus-seeding LPCs from aged BM were also able to compete with corresponding young LPCs even in subsequent *in vitro* culture (Fig. 4). These findings do not imply that LPCs do not age. In fact LPCs progressively lost their capacity to generate T-lineage cells with increasing age as demonstrated in a BMT setting, but they out-competed the ability of their niche cells to generate T-lineage with the passage of time (Fig. 5).

The mechanism by which young BM progenitor cells in the old BM-niches differentiate along the same pathway as old progenitor cells to make a myeloid skew (Fig. 1) is not clear. It is possible related to levels of reactive oxygen species (ROS) produced by BM niches since HSCs from a high oxygen milieu showed myeloid-skewed differentiation [37]. However, the central role of osteoblast-rich niches in this differentiation has been defined. This similar observation was reported [38], in which loss of osteoblasts in a gene knockout mouse model increased myelopoiesis. Another possible mechanism is hyperactivation of the mammalian target of rapamycin (mTOR) during aging [39, 40]. Although over-activation of mTOR in aging was found in stem cells [40], it is possible due to defects in stem cell regulation by the niche. A recently publication demonstrated the mechanism of calorie restriction (CR) on mammalian intestinal stem cells (ISC) during aging, in which CR, similar to Rapamycin administration, inhibits activity of mechanistic target of rapamycin complex 1 (mTORC1) in Paneth cells, a key constitute of the ISC niche. It results in promoting a more favorable stem-cell microenvironment, and then the changed microenvironment normalizes the stem cells [41].

Interestingly, when old BM progenitor cells were introduced into young mice, the young BM-niche did not rejuvenate differentiation profile of the old progenitor cells along the same pathway as young LPCs [23]. However, when old LPCs were recruited into young thymic niches *in vivo* (within KCT environment), the old LPCs were then directed to differentiate along the same differentiation pathway as young LPCs (Fig. 2). We believe this may be accounted for in two ways. 1) The BM of aged mice may contain both defective as well as functional hematopoietic progenitors, but only the functional progenitors are able to colonize the thymus. Thus, although aged BM has a reduced functional stem cell pool, there is still sufficient population of individual normal functional LPCs on a per cell basis [21] in aged mice. Because the thymic niche is gated [42] and number of the LPC recruitment is limited [an estimated ~10-100 new progenitor cells enter the young adult mouse thymus per day [43, 44]], the normal functional LPCs from aged BM seeding fetal thymus *in vivo* should be sufficient to provide the daily thymus recruitment requirement. Although the young functional BM stem cell pool is larger than the aged one, the total number of thymus-seeding LPCs from both pools should be the same at any given time. Therefore, grafted fetal thymic lobe-recruited LPCs from both aged and young mice are similar and produce the same number of T-lineage cells.

Nevertheless, when *in vitro* isolated aged LPCs were introduced into the young BM microenvironment via conventional BMT, and allowed to co-exist with young LPCs [10, 12, 45, 46], the aged LPCs are unable to compete, while in a cKCT microenvironment they are able to compete with young LPCs. This finding may be accounted for by 2) the reduced transplant efficacy of aged BM progenitors. For example, when removing aged BM progenitors from osteoblastic niches, they showed decreased adhesion to stroma [31]. *In vitro* manipulation of HSCs has been suggested to undergo replicative stress [47] or reduction of transplant efficiency [30]. Therefore, the defect in LPCs is only encountered with transplanted aged BM cells in conventional BMT [12], while this is not observed in physiological thymus-seeding aged BM cells (Figs. 2, 3, and 4) [1, 34]. Thus aged BM progenitors are not likely to have an intrinsic defect, but possess the stress from aged microenvironment, since it can be avoided by the *in vivo* collection (Figs. 2, 3, and 4).

Although LPCs/HSCs are not exempt from aging [48, 49], their aging occurs at a much slower rate than the aging of their niche cells, as measured by comparing the competence of thymic T-lymphopoiesis in a time-course manner based on BM progenitor ages or niche ages in a IL7R^−/−^ host BMT microenvironment (Fig. 5). Cells of both hematopoietic and non-hematopoietic origin undergo age-related deterioration from replicative stress and epigenetic changes [50] that influence DNA integrity [48], or exhaustion of their stem cell pools. However, during the process of natural aging, LPCs/HSCs outlive their niche cells, as evidenced by the onset in reduction in thymic T-lymphopoiesis of aged BM progenitors when repopulating the non-irradiated young IL7R^−/−^ thymus (at the age of 22 months). Concurrently, the niches lose their ability to support the production of functional T cells from the repopulating young BM progenitors in the same setting by “middle age” (~12 months of age in the mice).

In conclusion, the experiments in this study employed comprehensive *in vivo* and *in vitro* models to answer several pertinent questions. Which cellular component, HSC itself or HSC niche cell, determines HSCs to take age-related myeloid-skew developmental profile? Can the aged BM-niche influence competence of young BM progenitors to generate T-lineage cells by providing signals that are different from those provided by young niche cells? Why aged LPCs, derived from isolated BM progenitors, cannot compete with their young counterparts in a conventional BMT environment, while they can do so using *in vivo* collected natural thymus-seeding LPCs? Which cell type (LPCs or niche cells) primarily develops age-related inability in T-cell development in a time-course manner? We have answered these questions by showing clear evidence that the dominant and primary defect arising from aging of T-lymphopoiesis lies in a dysfunction of the niche cells [51] rather than in the T-cell progenitor pools. Further exploration of these issues will provide the foundation for gene-, pharmaceutical-, and stem-cell-based therapies by focusing on the right cellular targets and optimizing the timing for rejuvenation of reduced T-lymphopoiesis in order to treat aging-related onset of T-lymphocyte deficiency.

## METHODS

### Murine models and animal care

Mice from the C57BL/6 genetic background that expressed CD45.2^+^ or CD45.1^+^ congenic markers on the hematopoietic cell surface were used. Young (±2-month), early middle-aged (6~9-month), middle-aged (±12-month) and aged (over 18-month) (ages indicated in each figure) wild-type (WT), IL7R^−/−^ mice [26], and RAG^−/−^ fetal mice were originally purchased from the National Cancer Institute, National Institute for Aging /National Institutes of Health (NIH) (Bethesda, MD), and Jackson Laboratory (Bar Harbor, ME), respectively, and bred in our SPF animal facility. E15 indicates fetal mouse at embryonic day-15. All animal experiments were in compliance with the protocols approved by the Institutional Animal Care and Use Committee of the University of Texas Health Science Center at Tyler and the University of North Texas Health Science Center at Fort Worth, in accordance with guidelines of the NIH, USA.

### Comparison of myeloid vs. lymphoid fates in age-associated BM microenvironments

Erythrocyte-depleted BM cells (RBC lysing buffer from Sigma), from a pool of young CD45.1^+^ WT donor mice, were retro-orbitally transplanted (BMT) into 2- and 18-month-old CD45.2^+^ IL7R^−/−^ host mice (2 × 10^6^ cells/mouse). The donor LPCs [donor CD45.1^+^, lineage-negative, host CD45.2-negative, ckit^+^, andSca1^+^ BM population, i.e. LSK cells [52]] were sorted (FACS-Aria, Supplemental Fig. S1) from the young or old CD45.2^+^ IL7R^−/−^ hosts, 5 weeks after the BMT. These cells were cultured on an 80% confluent OP9-DL1 (kindly provided by Dr. J.C. Zuniga-Pflucker)[33] stromal cell monolayer (~2000 sorted donor LSK cells per culture well), in presence of Flt3 ligand (5 ng/ml) and IL-7 (5 ng/ml) (R&D). On the culture day 7, non-adherent cells were transferred to a fresh OP9-DL1 monolayer, and after 14 days in culture the cells were harvested and analyzed via flow cytometry for the differentiation of Mac1^+^ and Thy1.2^+^ cells derived from these sorted LSK cells (workflow in Fig. 1A).

### Kidney capsule transplantation (KCT)

Single-host KCT was performed as previously described [1] with the following modifications: (i) In group one the grafted fetal thymic lobes prior to KCT were treated with dGUO (2’-Deoxyguanosine, Sigma) for 5 days in fetal thymic organ culture (FTOC) for depleting hematopoietic cells in the lobes, while in group two the grafted fetal thymic lobes were intact prior to KCT; (ii) Thymocyte numbers and thymocyte differentiation profiles in the grafted fetal thymic lobes were monitored over time (1, 2, 3, and 4 weeks after the KCT). For cross KCT (cKCT), RAG^−/−^ mouse (CD45.1^+^CD45.2^+^ double congenic marker positive) E15 fetal thymic lobes from same littermate were subjected to KCT into first hosts of young (~2-month) CD45.1^+^ or aged (~22-month) CD45.2^+^ WT mice for 5 days. And then, cKCT was performed by transplanting the fetal thymic lobes carrying seeded LPCs from the first hosts into age-reversed and CD45-subtype-reversed second hosts to recruit natural thymus-seeding LPCs from the second hosts for additional 5 days. The thymic lobes contain young and aged (group-1) or aged and young (group-2) LPCs were harvested and cultured *in vitro* in 95% oxygen FTOC system [53] for ~7 days to expand the recruited LPCs before flow cytometric analysis (detailed workflow is shown in supplemental Fig. S2).

### Competitive reconstitution of natural thymus-seeding young and aged LPCs through an in vivo recruitment

The dGUO-treated fetal thymic lobes (E15 CD45.1^+^CD45.2^+^RAG^−/−^) were transplanted under the kidney capsules of young (~2-month) WT (CD45.1^+^) and aged (22-month) WT (CD45.2^+^) host mice, respectively, to recruit natural thymus-seeding LPCs over a 7-day period. The CD4 and CD8 double negative (DN, with CD3^−^) thymocytes from the grafted thymic lobe were then sorted by negative selection using magnetic beads (IMag^TM^ streptavidin particles, BD bioscience). The sorted cells (1:1 ratio of young and aged sources ~5000 cells each) were loaded on 80% confluent monolayers of OP9-DL1 stromal cells [33]in 12-well plates and culture in the presence of exogenously supplied IL-7 (5ng/ml) and Flt3 ligand (5ng/ml) (R&D). On the 5^th^ day of the culture non-adherent cells were transferred to a fresh OP9-DL1 monolayers, and ten days after the co-culture, the cells were isolated and analyzed by flow cytometry for CD45.1 vs. CD45.2, and CD4 vs. CD8 cell populations (workflow in Fig. 4A).

### Competence of BM progenitors or their niche cells of different ages to generate T cells

Experiment-A: to test the competence for thymic T-lymphopoiesis by BM progenitor cells from various ages, BM cells were obtained from CD45.2^+^ WT mice of ~2-, 8-, 12-, 18-, and 22-months of ages. About 2 × 10^6^ erythrocyte-depleted BM cells were retro-orbitally injected into non-irradiated CD45.1^+^ young IL7R^−/−^ host mice to reconstitute the donor T-cell pool in the host thymus. Experiment-B: to test competence for thymic T-lymphopoiesis in LPC niches (both BM and thymic niches) of different ages, ~2 × 10^6^ erythrocyte-depleted BM cells from a pool of young CD45.1^+^ WT mice were retro-orbitally injected into ~2-, 6-, 8, 12~13-, and 18-month-old non-irradiated CD45.2^+^ IL7R^−/−^ host mice. Five weeks after the injection, the donor-derived thymic T-lymphopoiesis was analyzed by flow cytometry (workflow in Fig. 5A).

### Flow cytometric assay

Cell counting was carried out using the BioRad TC10^TM^ automated cell counter or a hemacytometer. For flow cytometric analysis, thymocytes isolated with a cell-strainer were blocked with anti-FcR (clone 2.4G2) to diminish nonspecific binding, and then stained with combinations of fluorochrome-conjugated antibodies (as indicated in each figure). Data were acquired by a dual-laserFACS-Calibur system and analyzed with CellQuest and FlowJo software.

### Statistics

Statistical significance was analyzed by unpaired Student's *t*-test with Welch's correction (if variances shown unequal) or Mann-Whitney test (if data shown non-parameter). Linear regression is used to fit straight lines for the two groups. By using a dummy variable to fit two regression lines in a single equation, the equality of the two slopes is tested based on a t-test. Differences were considered statistically significant at values of *p* < 0.05.

## SUPPLEMENTARY FIGURES



## Abbreviations

BMT: bone marrow transplantation
FTOC: fetal thymic organ culture
KCT: kidney capsule transplantation
LPC/HSC: lympho-hematopoietic progenitor cell/hematopoietic stem cell
LSK: lineage-negative, Sca1-positive, cKit-positive population
TEC: thymic epithelial cell
WT: wild type
