# Supplementary material for: Aging induced decline in T-lymphopoiesis is primarily dependent on status of progenitor niches in the bone marrow and thymus
Source: Aging (Albany NY). 2012 Oct 8;4(9):606–19. doi: 10.18632/aging.100487 (PMC3492225; doi:10.18632/aging.100487)
Supplement: Supplementary file 1 [file aging-04-606-s001.pdf]

49. Marciniak-Czochra A, Stiehl T and Wagner W. Modeling of replicative senescence in hematopoietic development. Aging (Albany NY). 2009; 1:723-732.

50. Issa JP. Age-related epigenetic changes and the immune system. Clin Immunol. 2003; 109:103-108.

51. Wilson A and Trumpp A. Bone-marrow haematopoietic-stem-cell niches. Nat Rev Immunol. 2006; 6:93-106.

52. Schwarz BA and Bhandoola A. Circulating hematopoietic progenitors with T lineage potential. Nat Immunol. 2004; 5:953-960.

53. Su DM, Wang J, Lin Q, Cooper MD and Watanabe T. Interferons alpha/beta inhibit IL-7-induced proliferation of CD4- CD8- CD3- CD44+ CD25+ thymocytes, but do not inhibit that of CD4- CD8- CD3- CD44- CD25- thymocytes. Immunology. 1997; 90:543-549.

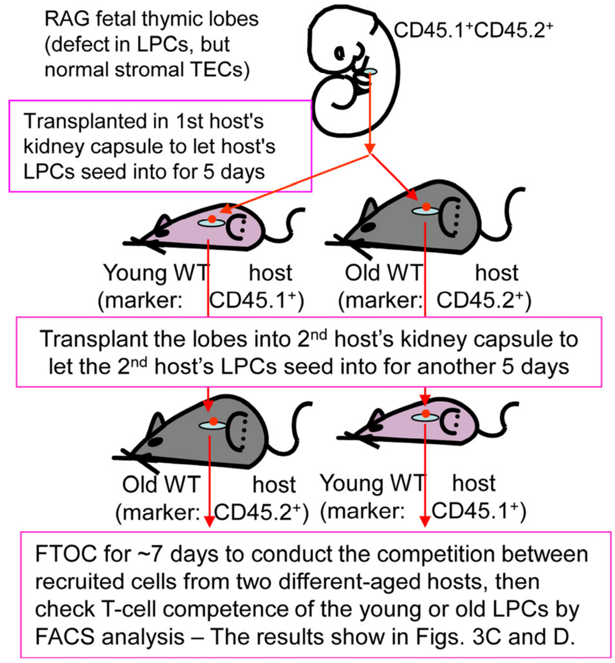

Supplementary Fig. S2. Schematic workflow of the *in vivo* competitive repopulation assay via a cross kidney capsule transplantation setting. Schematic representation of the *in vivo* competitive cross kidney capsule transplantation (cKCT) assay to accumulate both young and old natural thymus-seeding cells in the same grafted fetal thymic lobes *in vivo* and to conduct the competitive *in vitro* assay in a fetal thymic organ culture (FTOC) setting.

SUPPLEMENTARY FIGURES

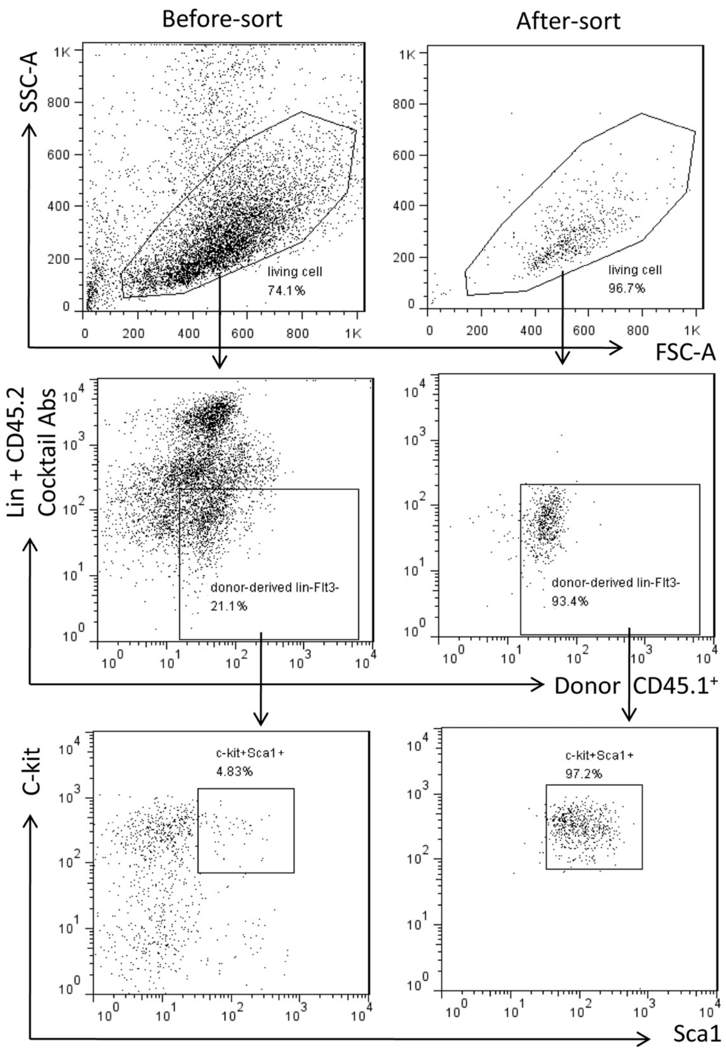

Supplementary Fig. S1. Purification of LSK cells before and after FACS-sort. Top panels show live gates; middle panels show lineage and host-CD45.2 (cocktail antibodies) negative and donor-CD45.1$^+$ gate; Bottom panels show Sca-1$^+$ and c-Kit$^+$ gate in Lin$^-$ and host-CD45.2$^-$, and Donor-CD45.1$^+$ population. Left panels show BM cells before sorting; Right panels show BM cell after sorting.

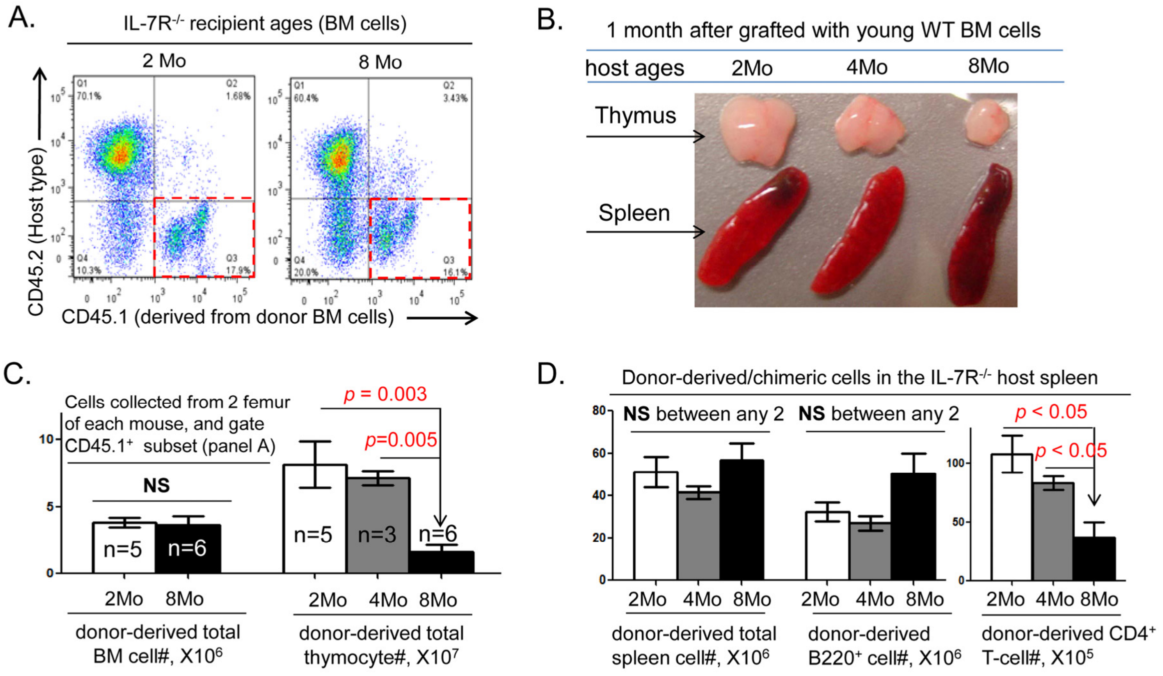

Supplementary Fig. S3. Integrated analysis of CD45.1/ CD45.2 chimerism pre- and post-thymus. One month after BMT of CD45.1^{+} WT young BM progenitors into CD45.2^{+} IL7R$^{-/-}$ recipients of a range of ages, donor-type BM cells, thymocytes, splenic B and T cells were analyzed. (**A**) Representative result of donor-derived CD45.1^{+} BM cells (red boxes) in CD45.2^{+} IL7R$^{-/-}$ hosts of a range of ages; (**B**) Image shows size of the thymus and spleen from IL7R$^{-/-}$ grafted with same WT young BM progenitors; (**C**) Left: donor-derived total BM cell #, cell counting from 2 femur, multiplying panel A % in red boxes; Right: a summary of donor-derived total thymocyte #. “NS = no significant.” (**D**) Donor-derived total spleen cell#, B cell# and CD4^{+} T-cell# in the recipients’ spleens of a range of ages. (In this supplemental experiment, recipient IL7R$^{-/-}$ mouse number: 2Mo = 5; 4Mo = 3; 8Mo = 6).

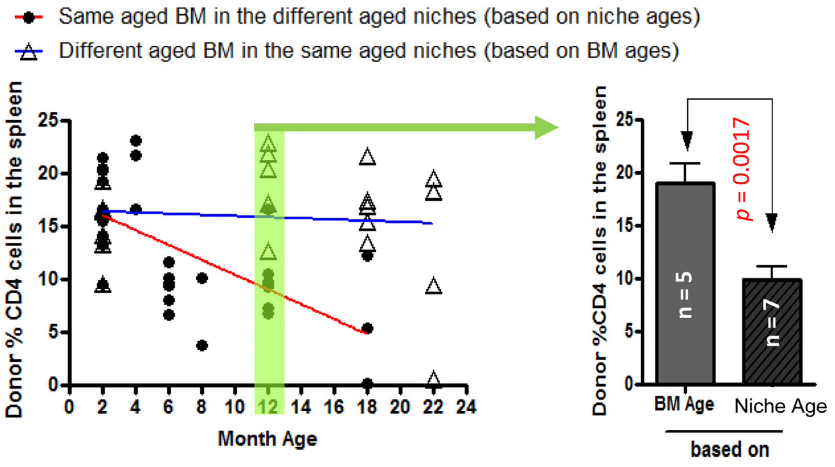

Supplementary Fig. S4. Comparing engraftment capacity to repopulate periphery based on LPCs’ age or niche cells’ age in a CD45 subtype-mismatched BMT model with an unirradiated IL7R$^{-/-}$ mouse as a host.
Left Panel: A linear regression assay of % CD4^{+} T cells in the spleen, derived from donor WT BM cells of different ages in young IL7R$^{-/-}$ host niches (based on donor BM progenitor ages, blue line) and derived from young donor WT BM cells in IL7R$^{-/-}$ host niches of different ages (based on recipient niche ages, red line). Test for equal slopes for the blue and red lines a (2-sided) p-value of 0.009 (significantly different). Each triangle and round represents one recipient animal. Right panel: Donor-derived % CD4^{+} T cells in the spleen of 12-month-old donor WT BM cells in young IL7R$^{-/-}$ host niches (left bar) and young donor WT BM cells in 12-month-old IL7R$^{-/-}$ host niches (right bar). Data show mean ± SEM in bar graph, n = IL7R$^{-/-}$ host animal number.
